# Incidence of Second Malignancies Among Children Treated for Cancer With Radiotherapy in Egypt

**DOI:** 10.1002/cnr2.70542

**Published:** 2026-05-18

**Authors:** Charlotte L. Sackett, Mohamed S. Zaghloul, Ahmed Aldesouky, Alaa Elhaddad, Li Zhang, Chloe A. Teasdale, Amr S. Soliman

**Affiliations:** ^1^ Department of Population and Public Health Sciences, Keck School of Medicine University of Southern California California Los Angeles USA; ^2^ Department of Radiation Oncology, National Cancer Institute Cairo University & Children's Cancer Hospital Cairo Egypt; ^3^ Department of Radiation Oncology Children's Cancer Hospital Cairo Egypt; ^4^ Department of Pediatric Oncology, National Cancer Institute Cairo University & Children's Cancer Hospital Cairo Egypt; ^5^ Community Health and Social Medicine Department, CUNY School of Medicine The City College of New York New York New York USA; ^6^ Department of Epidemiology and Biostatistics, Graduate School of Public Health and Health Policy City University of New York (CUNY) New York New York USA

**Keywords:** chemotherapy, Egypt, LMIC, oncology, pediatric cancer, radiation, radiotherapy, secondary cancer, secondary malignancy, SMN

## Abstract

**Background:**

Survivors of childhood cancer (CCS) are at risk for secondary malignant neoplasms (SMNs): serious late effects of treatment. Radiotherapy (RT) and chemotherapy (CT) are possible causes of SMNs.

**Aims:**

To measure SMN incidence and characteristics among Egyptian CCS treated with RT, and to compare SMN timing and location based on RT field and CT type.

**Methods and Results:**

We performed a retrospective cohort study of patients under 18 years who received radiotherapy for primary cancer at the Children's Cancer Hospital, Cairo, Egypt, from 2009 to 2015, with follow‐up through 2021. We calculated cumulative incidence functions for second malignant neoplasm (SMN) development overall and by primary diagnosis, using the Fine and Gray method, both for all SMNs and for in‐field SMNs. We used the Wilcoxon rank‐sum test to compare time to SMN between patients with in‐field versus out‐of‐field SMNs and those who received alkylating versus non‐alkylating chemotherapy. Of 3132 patients (60.1% male; median age 6.2 years), 23 developed an SMN (11.1‐year cumulative incidence: 1.85%, 95% CI: 0.84%–3.57%). Eight (34.8%) of the 23 SMNs arose within the prior RT field (11‐year cumulative incidence: 2.71%, 95% CI: 1.32%–4.93%). Median time to SMN was longer for in‐field than out‐of‐field cases (7.9, IQR: 6.0–9.9 vs. 4.3 years, IQR: 2.7–5.6; *p* = 0.002). There was no significant difference in time to SMN by CT type.

**Conclusion:**

SMN incidence in this cohort matched rates in high‐income countries. Strong long‐term follow‐up is essential for CCS in Egypt and similar settings.

## 1 Introduction

Cancer is a leading cause of morbidity and mortality for children globally [1]. In 2022, the International Agency for Research on Cancer reported that 275 000 children aged 0–19 were diagnosed with cancer, and more than 100 000 died [2]. Advances in treatment have markedly improved survivorship for children with cancer in high‐income countries, with the United States reporting an increase in overall 5‐year survival from 10% to 85% over the past four decades [3, 4].

Despite these improvements, primary cancer treatment has also been investigated as a potential cause of secondary malignant neoplasms (SMNs), which are defined as new primary cancers that are histologically distinct from the initial tumor and develop after treatment for childhood cancer [5]. SMNs are the most frequent cause of non‐recurrence, health‐related death among 5‐year survivors of childhood cancer and contribute substantially to long‐term mortality, accounting for ~47.6% of non‐recurrence health‐related deaths across extended follow‐up [6]. Though SMNs can occur any time after an initial cancer diagnosis, the few available studies on long‐term outcomes among CCS have reported that SMNs are more likely to occur two or more decades after the original diagnosis [7].

Radiotherapy (RT), a treatment modality included in roughly 50% of all cancer regimens, is a recognized risk factor for SMN due to its mutagenic effect on deoxyribonucleic acid (DNA) [8, 9, 10]. Among Egyptian cancer patients ≤ 18 years, around 33% receive RT alone or together with other modalities [11]. In a study of American and Canadian CCS between 1970 and 1999, the cumulative incidence of SMN 30 years after initial diagnosis approached 11% for those who received RT for primary cancer.12 SMN incidence was also higher in CCS patients receiving RT than in those not receiving RT.12.

Chemotherapy (CT), which is used independently and in conjunction with RT in cancer treatment protocols, has also been shown to contribute to the risk of SMN due to its carcinogenic effect and potential interaction with underlying genetic factors [12]. Certain chemotherapies, such as alkylating agents, have a greater impact on the SMN rate, with one specific study observing a dose–response relationship between alkylating agents and SMN rate [13].

There is a dearth of information on survival and SMN incidence among CCS from the Middle East and North Africa (MENA) region, and few studies worldwide report evidence from CCS cohorts treated with radiation in the past 20 years. The objective of this study was to describe the incidence of SMN among CCS patients treated with RT in a large, well‐equipped regional pediatric cancer center in Egypt.

## 2 Methods

We conducted a retrospective cohort study of patients younger than 18 years of age who were diagnosed with and treated for a primary malignancy at Children's Cancer Hospital, Egypt (CCHE), Cairo, from January 2009 through December 2015, with follow‐up through December 2021. CCHE is a specialized pediatric oncology hospital in the MENA region, serving nearly half of all childhood cancer cases diagnosed in Egypt in 2019 [14]. Patients were eligible if they received at least one full RT course as part of their primary cancer treatment. Additional inclusion criteria were Egyptian residency and absence of a Stage V Wilms primary diagnosis. Stage V Wilms' tumors, either synchronous or metachronous, have the same pathology and are not second malignancies or metastases. There are a few Stage V Wilms tumors in this cohort; exclusion does not affect denominators or introduce bias due to the rarity of this diagnosis [15]. Ethical and administrative approvals were received from the Institutional Review Board of the University of Southern California and the Scientific Medical Advisory Committee of CCHE.

Demographic and clinical data were extracted from electronic medical records, including sex, province of residence, hospital registration date (date of entry for initial cancer diagnosis), age at registration, primary diagnosis, cancer site and stage, metastatic status, CT treatment, intent and dose of each RT course, SMN occurrence, and whether the SMN developed within the RT field. For this study, a secondary malignant neoplasm (SMN) refers specifically to a histologically distinct malignancy (i.e., unrelated to the recurrence or metastasis of the primary cancer) that develops after completion of primary cancer treatment. All SMN diagnoses were identified, histologically confirmed, and reviewed by treating physicians through chart review. The primary exposure of interest was the RT field location relative to the SMN site, classified as in‐field (SMN arising within the prior RT field) or out‐of‐field. The CT agent was classified as alkylating or non‐alkylating. Patients who received combination alkylating and non‐alkylating CT were classified into the alkylating group.

Per CCHE protocols, each patient was regularly followed by a clinician via telephone approximately every 6 months until they reached 23 years of age. The follow‐up time for each patient was measured from the hospital registration date to one of the following: SMN, death, loss to follow‐up (LTFU), or the end of study (August 31, 2021).

The primary outcome was time from first hospital registration to SMN diagnosis. Death prior to SMN was treated as a competing risk. Cancer recurrence was not used as a censoring event or competing risk; patients who experienced recurrence remained at risk for SMN and were followed to their primary endpoint. We estimated the cumulative incidence function (CIF) for SMN development using the Fine and Gray method, which accounts for the subdistribution hazard in the presence of competing risks [16]. CIFs were estimated overall and stratified by primary cancer diagnosis. We similarly estimated the CIF restricted to in‐field SMNs, overall, and by primary diagnosis. Cumulative incidence functions (CIFs) were estimated separately for hematologic and solid tumor SMNs, accounting for the competing risks of death and the opposing SMN type. Fisher's Exact Test was used to assess whether the distribution of SMN type (hematologic vs. solid tumor) differed significantly by RT field location (in‐field vs. out‐of‐field), restricted to patients who developed an SMN (*n* = 23). We used the Wilcoxon rank‐sum test to compare time to SMN between patients with in‐field versus out‐of‐field SMNs and between patients who received alkylating versus non‐alkylating CT. All analyses were performed using SAS version 9.4 (SAS Institute Inc., Cary, NC, USA). Statistical significance was set at *p* < 0.05.

## 3 Results

After excluding patients from outside Egypt (*n* = 112) and those with Stage V Wilms tumor (*n* = 29), a total of 3132 eligible patients treated at CCHE between 2009 and 2015 were included in the analysis, of whom the majority (60.1%) were male (Table 1). The median age at registration was 6.2 years (interquartile range (IQR) 3.5–10.7 years). Nearly half (48.2%) of patients were from lower Egypt, followed by Greater Cairo (31.7%) and Upper Egypt (20.1%). The most frequent primary cancer diagnoses were grouped as “other tumors” (37.0%), which included acute leukemias and epithelial, renal, and brain tumors (30.1%). Abdomen and pelvis (27.9%) and head and neck (20.3%) were the most common cancer sites. For the majority (57.6%) of patients, cancer stage was not applicable; among those with a stage, 62.8% and 37.2% were diagnosed with Stages III–IV and Stages I–II, respectively. Metastatic status was not relevant for 28.5% of patients; among those with a known status, the majority (68.2%) were metastatic (Table 1).

**TABLE 1:** Characteristics of pediatric patients < 18 years of age who received radiotherapy at Children's Cancer Hospital, Egypt (CCHE) between 2009 and 2015 (*N* = 3132).

	*N*	%
	3132	100.0
Demographics		
Sex		
Male	1881	60.1
Female	1251	39.9
Region^a^		
Lower Egypt	1509	48.2
Greater Cairo	992	31.7
Upper Egypt	630	20.1
Registration year		
2009	271	8.7
2010	293	9.4
2011	398	12.7
2012	452	14.4
2013	477	15.2
2014	605	19.3
2015	636	20.3
Age at registration (years)^b^, median (IQR)	6.2	3.5–10.7
0–4	1086	34.7
5–9	1080	34.5
10–14	656	20.9
15–18	310	9.9
Diagnosis		
Primary Diagnosis		
Other tumors	1159	37.0
Liver tumors	6	0.2
Nasopharyngeal carcinoma	42	1.3
Neuroblastoma	328	10.5
Retinoblastoma	63	2.0
Bone tumors	248	7.9
Brain tumors	944	30.1
Soft tissue sarcoma	342	10.9
Cancer site^c^		
Head and neck	635	20.3
Chest	76	2.4
Abdomen and pelvis	872	27.9
Limbs	170	5.4
Head, neck, and chest	47	1.5
Chest, abdomen, and pelvis	118	3.8
Head, neck, chest, abdomen, and pelvis	93	3.0
Central nervous system	1120	35.8
Cancer stage (among those with data)^d^		
I–II	492	37.2
III–IV	830	62.8
Cancer stage not applicable	1796	57.6
Metastatic status (among those with data)^e^		
No	1524	68.2
Yes	712	31.8
Metastatic stage not applicable	892	28.5
Treatment characteristics		
Intent of first radiotherapy course		
Curative	2615	83.5
Salvage	142	4.5
Palliative	375	12.0
Chemotherapy^f^		
No	481	15.4
Yes	2649	84.6

Abbreviation: RT, radiotherapy.

^a^ One patient did not have region data available.

^b^ One patient did not have age at registration data available.

^c^ One patient did not have cancer site data available.

^d^ Fourteen patients did not have cancer stage data available.

^e^ Four patients did not have metastatic status data available.

^f^ Two patients did not have chemotherapy data available.

Over the study period, median follow‐up time was 5.7 years (IQR 1.8–7.9; 95% CI: 5.60–5.85). A total of 1210 patients (38.6%) died without an SMN and were treated as competing events, and 1899 (60.6%) were censored at LTFU or end of study (August 31, 2021). Of the 1210 patients who died without an SMN, 386 (31.9%) died within the first year of follow‐up.

**FIGURE 1:**
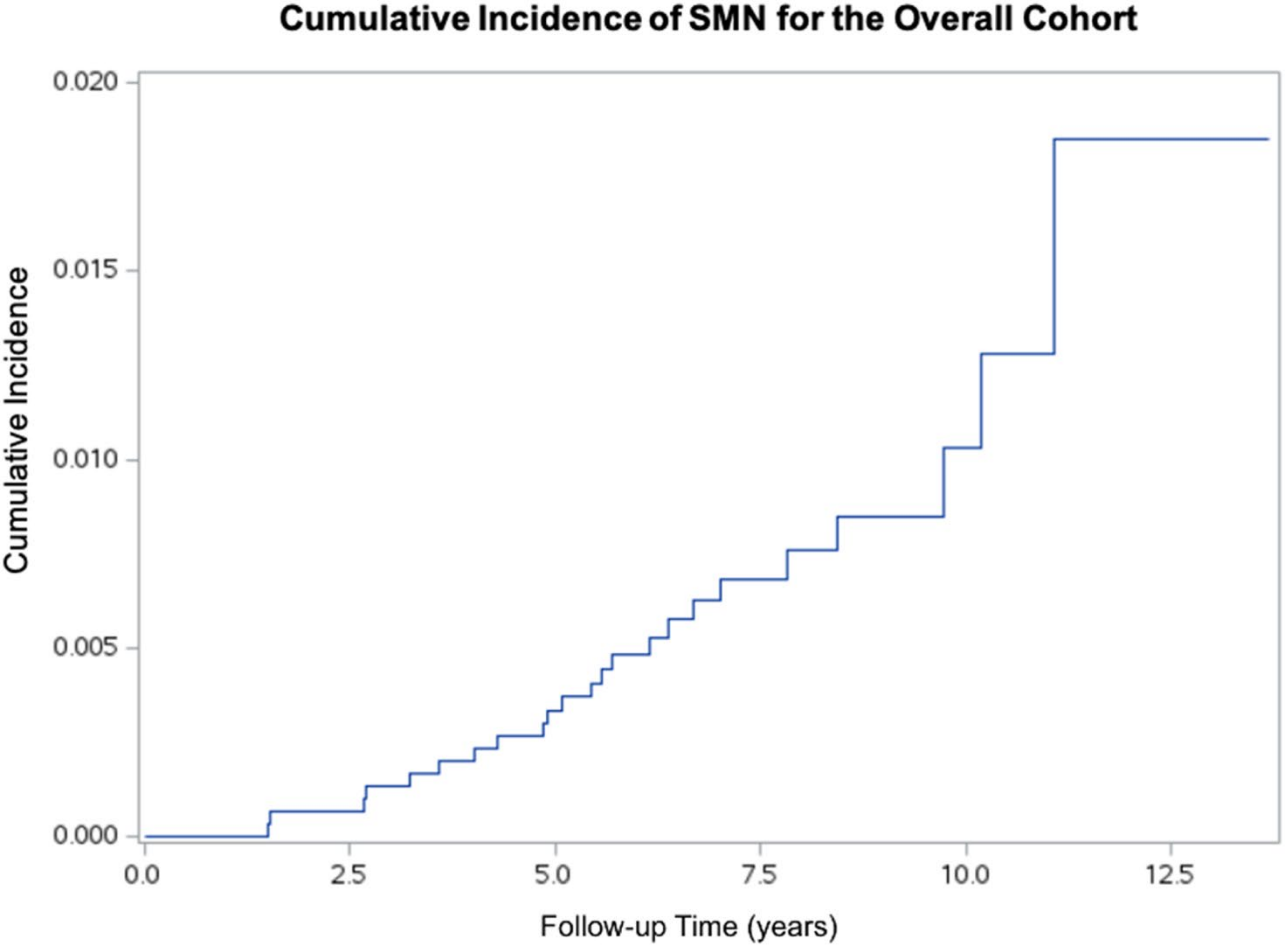
Cumulative incidence of secondary malignant neoplasms among children with cancer treated at Children's Cancer Hospital, Egypt (CCHE) between 2009 and 2015 (*n* = 3132).

Twenty‐three (0.7%) CCS developed an SMN; the cumulative incidence at 11 years was 1.85% (95% CI: 0.84%–3.57%) (Figure 1). All CCS who developed SMNs received CT in addition to RT; 17 (73.9%) received alkylating CT, and 6 (26.1%) received non‐alkylating CT. Of the 17 SMNs that occurred among those who received alkylating CT, 5 (29.4%) were AML, 4 (23.5%) were MDS, 4 (23.5%) were osteosarcomas, 2 (11.8%) were glioblastomas, 1 (5.9%) was neuroblastoma, and 1 (5.9%) was a renal cell carcinoma. Of the six SMNs that occurred among those who received non‐alkylating CT, there were 2 (33.3%) large B‐cell diffuse malignant lymphomas, and the remaining were 1 (16.7%) each of alveolar rhabdomyosarcoma, alveolar soft part sarcoma, Ewing sarcoma, and papillary carcinoma of the thyroid.

**FIGURE 2:**
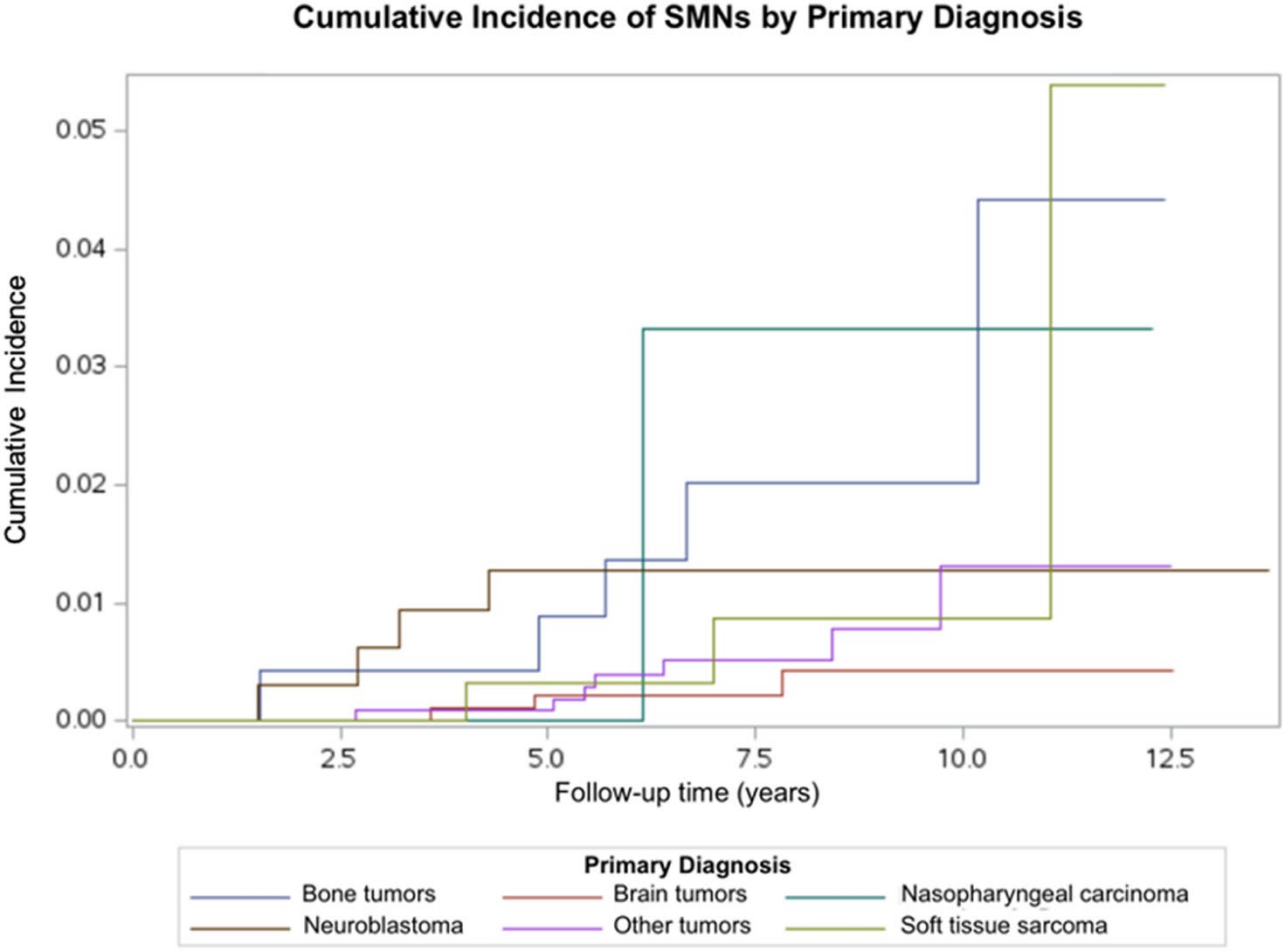
Cumulative incidence of secondary malignant neoplasms among children with cancer treated at Children's Cancer Hospital, Egypt (CCHE) between 2009 and 2015 by primary diagnosis (*n* = 3132).

Cumulative SMN incidence varied by primary diagnosis (Figure 2). The highest 11‐year CIFs were observed among patients with soft tissue sarcomas (CIF: 5.39%, 95% CI: 0.58%–19.13%), followed by bone tumors (CIF: 4.42%, 95% CI: 1.09%–11.58%) and nasopharyngeal carcinomas (CIF: 3.32%, 95% CI: 0.23%–14.81%), though CIs were wide given the small number of events within each group. Brain tumor survivors had the lowest observed cumulative incidence (CIF 0.43%, 95% CI: 0.11%–1.26%) at the last observed event time.

**FIGURE 3:**
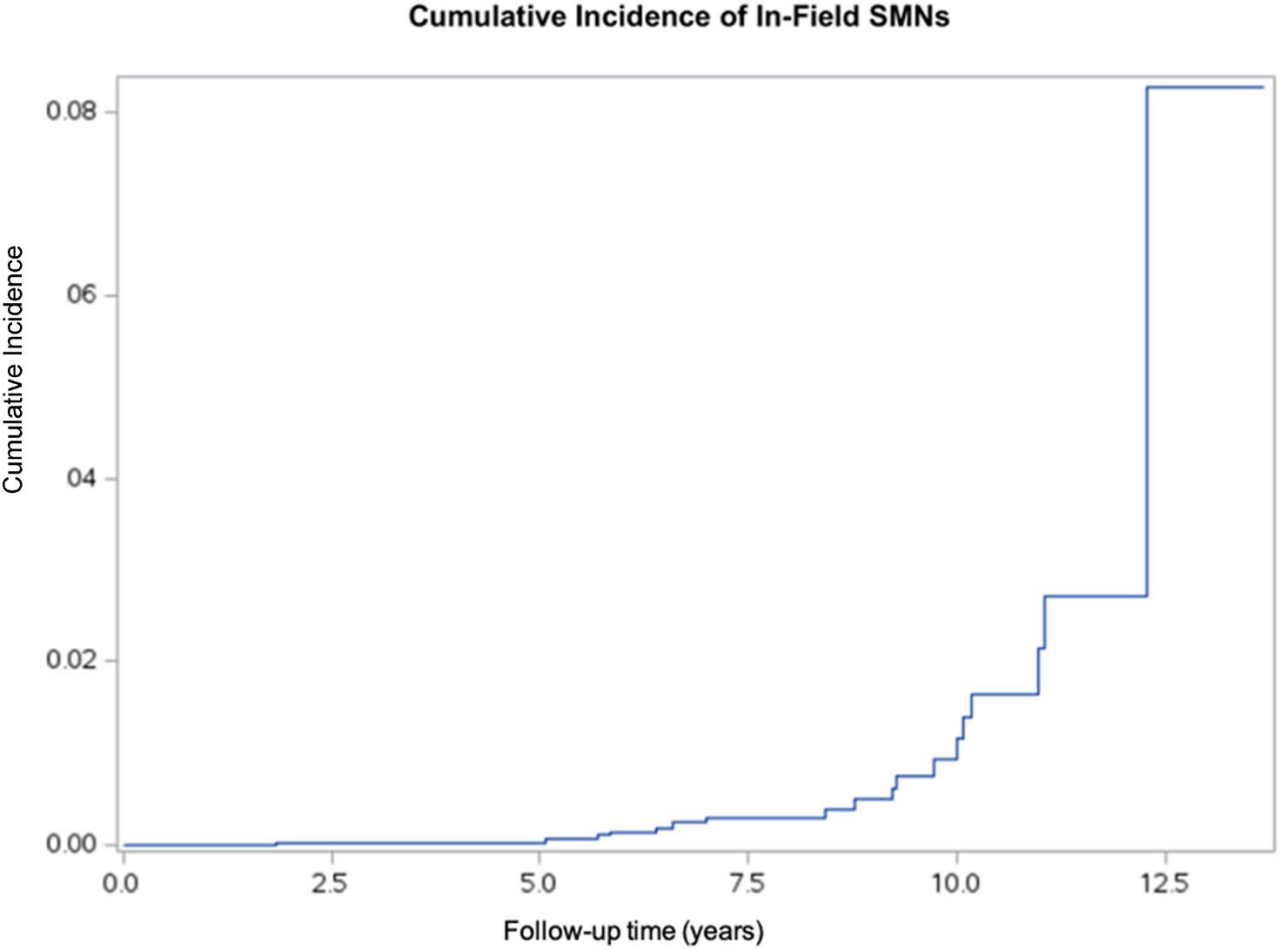
Cumulative incidence of in‐field secondary malignant neoplasms among children with cancer treated at Children's Cancer Hospital, Egypt (CCHE) between 2009 and 2015 (*n* = 3132).

Of the 23 SMNs, 8 (34.8%) arose within the prior RT field. The 11‐year cumulative incidence of RT‐field SMNs was 2.71% (95% CI: 1.32%–4.93%) (Figure 3). Of the SMNs that occurred within the RT field, half were osteosarcomas, and the remaining were 1 (12.5%) each of alveolar soft part sarcoma, rhabdomyosarcoma, Ewing sarcoma, and thyroid tumor. Among those that occurred outside of the RT field, there were 5 (35.7%) cases of acute myeloid leukemia (AML), 4 (28.6%) cases of myelodysplastic syndrome (MDS), 2 (14.3%) cases of glioblastoma, 2 (14.3%) cases of lymphoma, and 1 (7.1%) case of a kidney tumor.

There was no statistically significant difference in time to SMN between patients who received alkylating versus non‐alkylating CT (*p* = 0.15). Median time to SMN was 4.8 years (IQR 3.2–8.4 years) among those who received alkylating CT (*n* = 17) and 6.0 years (IQR: 5.4–8.4 years) among those who received non‐alkylating CT (*n* = 6), though this comparison should be interpreted cautiously given the small number of events in each group.

Time to SMN differed significantly by RT field location (*p* = 0.002). In‐field SMNs occurred significantly later than out‐of‐field SMNs, with a median time to SMN of 7.9 years (IQR 6.0–9.9 years) versus 4.3 years (IQR 2.7–5.6 years), respectively. This pattern is consistent with the longer latency typically observed for radiation‐induced solid tumors compared to CT‐related hematologic malignancies, which tend to manifest earlier after treatment.

**FIGURE 4:**
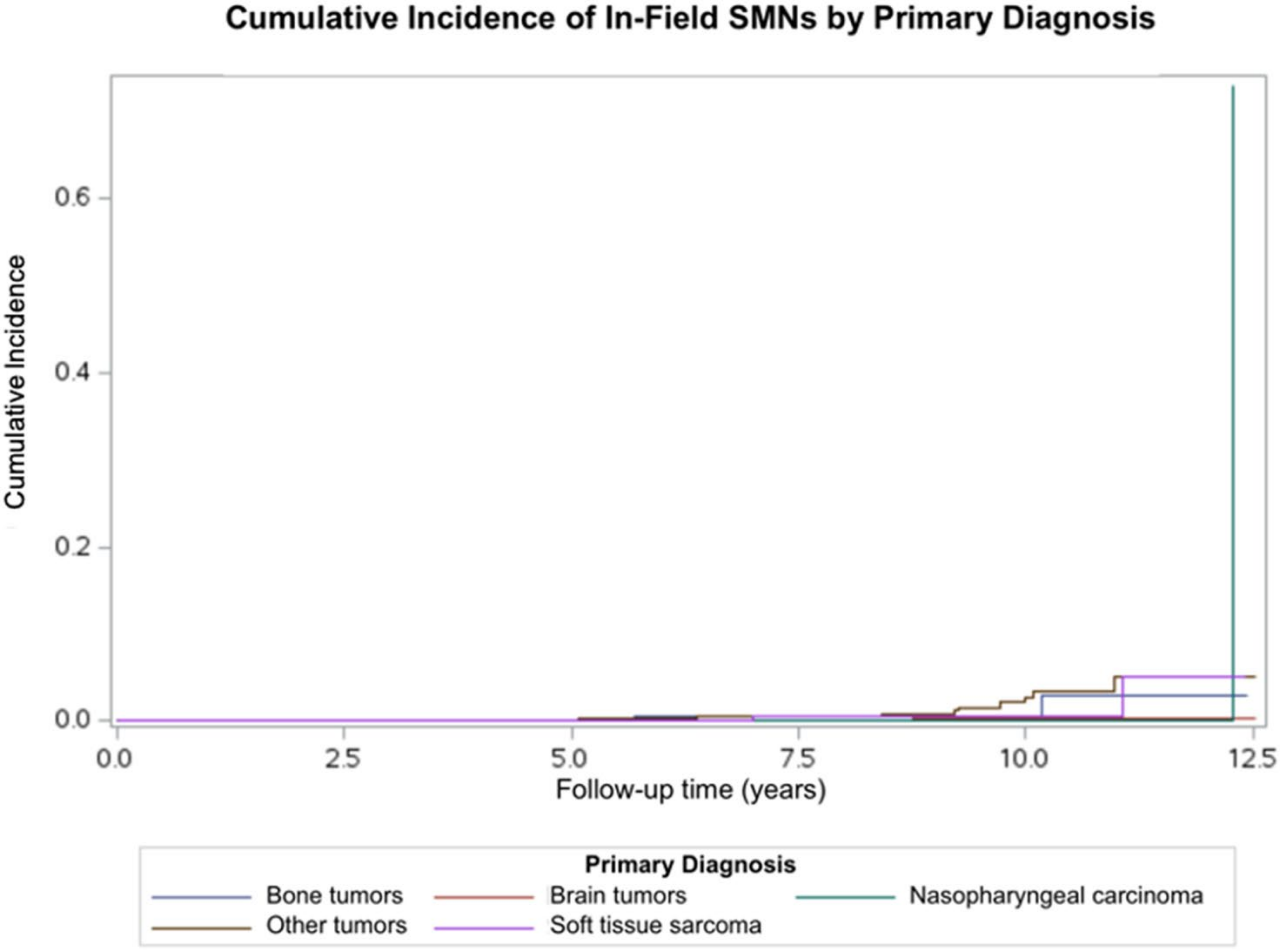
Cumulative incidence of in‐field secondary malignant neoplasms among children with cancer treated at Children's Cancer Hospital, Egypt (CCHE) between 2009 and 2015 by primary diagnosis (*n* = 3132).

When stratified by primary diagnosis, RT‐field SMNs were observed among patients with bone tumors, brain tumors, nasopharyngeal carcinoma, soft tissue sarcoma, and other tumors; no RT‐field SMNs occurred among survivors of neuroblastoma, retinoblastoma, or liver tumors. The 11‐year cumulative incidence of RT‐field SMN was highest among survivors of other tumors (5.09%, 95% CI: 2.06%–10.19%), followed by soft tissue sarcoma (5.08%, 95% CI: 0.46%–19.18%), though no RT‐field SMNs in soft tissue sarcoma survivors were observed before year 7. Bone tumor survivors had a cumulative incidence of 2.88% at 10.2 years (95% CI: 0.34%–10.88%), and brain tumor survivors had the lowest observed RT‐field SMN incidence (0.29%, 95% CI: 0.03%–1.55%), based on a single event at 8.8 years with no subsequent events through the end of follow‐up (Figure 4). All diagnosis‐specific estimates carry wide confidence intervals reflecting the small number of events within each stratum.

**FIGURE 5:**
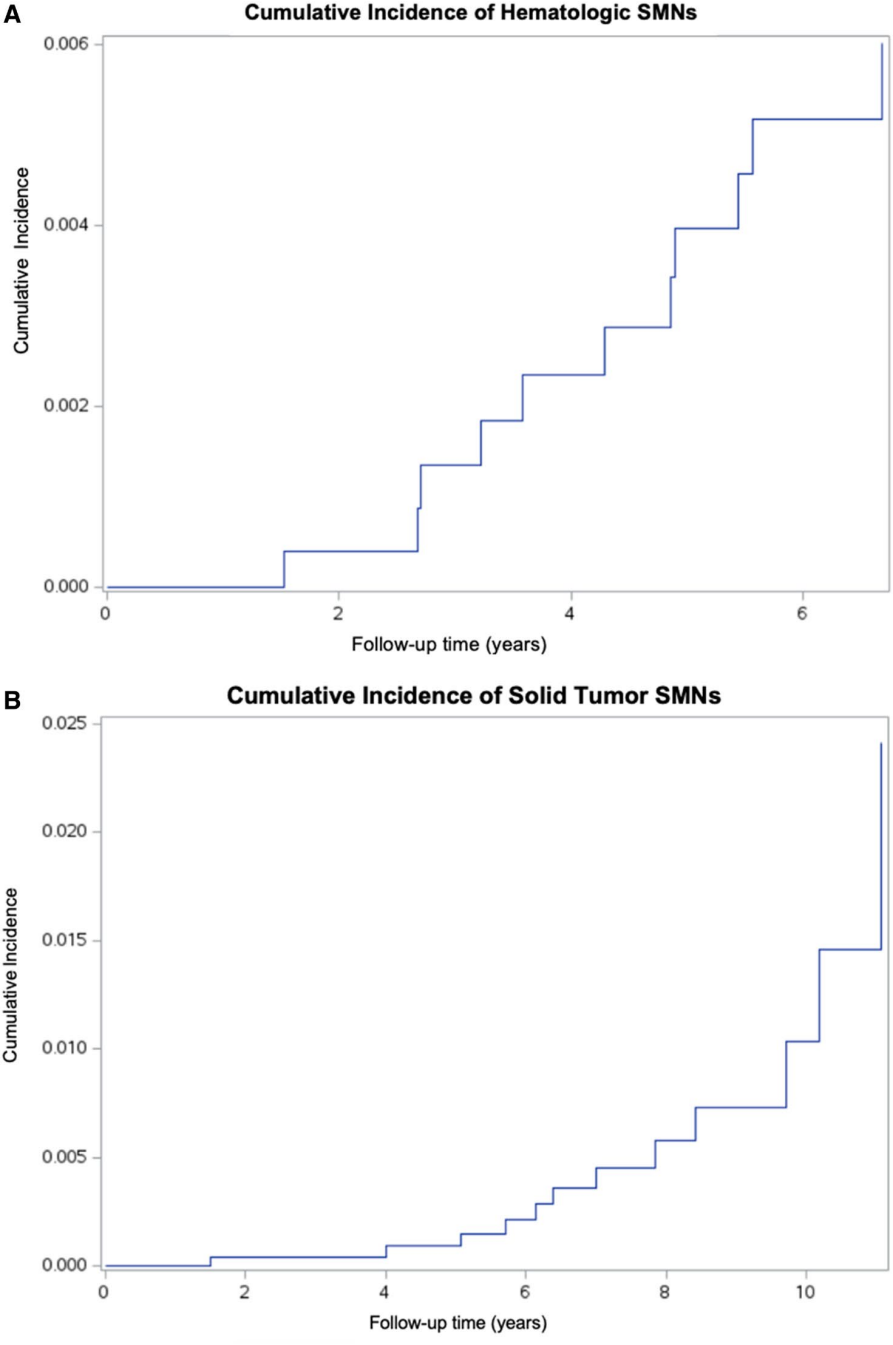
Cumulative incidence of hematologic secondary malignant neoplasms (A) and solid secondary malignant neoplasms (B) among children with cancer treated at Children's Cancer Hospital, Egypt (CCHE) between 2009 and 2015 (*n* = 3132).

Of the 23 SMNs, 11 (47.8%) were hematologic malignancies and 12 (52.2%) were solid tumors. The cumulative incidence of solid tumor SMNs at 11 years was 2.42% (95% CI: 1.05%–3.78%), while the cumulative incidence of hematologic SMNs was 0.60% (95% CI: 0.25%–0.96%) at the time of the last observed hematologic event (6.7 years), with no further hematologic SMNs observed thereafter (Figure 5). The distribution of SMN type differed significantly by RT field location (*p* = 0.0013); all 8 in‐field SMNs were solid tumors (100%), while 11 of 15 out‐of‐field SMNs (73.3%) were hematologic malignancies.

## 4 Discussion

In this study of Egyptian CCS treated with RT between 2009 and 2015, nearly 2% developed an SMN 11 years after initial treatment; among those with an SMN, more than a third occurred within the field of RT. Though other studies that determined SMN incidence among CCS used differing follow‐up periods, our estimate of a 2% cumulative incidence of SMN is consistent with what would be expected given our follow‐up duration. In a Canadian CCS cohort that was diagnosed with a first cancer between 2000 and 2015, 0.7% developed an SMN within the first 5 years following initial diagnosis [17]. Another study of American and Canadian CCS treated with RT between 1970 and 1999 reported SMN cumulative incidence of ~11% 30 years' post‐initial diagnosis [13]. There are a few other estimates of SMN incidence among CCS in the first decade post‐treatment, and none from the past 30 years.

Another important finding is that 35% of SMNs occurred within the RT field. In contrast to studies of single‐tumor‐type cohorts, such as irradiated medulloblastoma patients, in which nearly all SMNs arose within the RT field [18], our mixed‐diagnosis cohort includes a substantial proportion of hematologic malignancies occurring outside the field. These—specifically AML and MDS—are not plausibly attributed to localized RT exposure, but are instead well‐recognized sequelae of alkylating CT [12]. The apparent predominance of out‐of‐field SMNs therefore likely reflects the combined carcinogenic burden of multimodal therapy rather than a lower RT‐attributable risk. Several additional factors may help explain the comparatively lower proportion of in‐field SMNs. First, dose heterogeneity across the RT field means that not all irradiated tissue receives a uniformly carcinogenic dose—regions receiving intermediate or low scatter doses may carry mutagenic potential without reaching the threshold typically associated with in‐field solid tumor induction, potentially displacing some RT‐attributable SMNs to out‐of‐field classifications [19]. Second, surveillance bias may play a role; patients who received RT to anatomically visible or symptomatically accessible sites may be more likely to have SMNs detected outside the field incidentally during routine follow‐up, whereas slow‐developing in‐field solid tumors may remain subclinical within our follow‐up window. Third, the high competing risk of mortality in this cohort (nearly 40% of patients died during follow‐up) disproportionately truncates long‐term observation, and given that in‐field solid tumors such as sarcomas have a significantly longer latency (mean 7.5 years in our cohort), many RT‐attributable SMNs may simply not have had sufficient time to manifest. Finally, the heterogeneity of cancer types included in this study means that RT field locations, volumes, and doses varied considerably across patients, likely diluting the in‐field signal relative to studies of more homogeneous, heavily irradiated populations such as medulloblastoma.

When SMNs were classified by type, a highly significant difference in distribution by RT field location was observed (*p* = 0.0013), with all in‐field SMNs being solid tumors and nearly three‐quarters of out‐of‐field SMNs being hematologic malignancies. This pattern is consistent with the established biology of treatment‐related malignancy: RT‐induced carcinogenesis predominantly manifests as solid tumors arising within or adjacent to the irradiated field, while hematologic malignancies—particularly AML and MDS—are well‐recognized sequelae of alkylating chemotherapy and are not plausibly attributable to localized RT exposure [20]. The divergent CIF trajectories further illustrate this distinction, with solid tumor SMNs accruing gradually across the full follow‐up period and hematologic SMNs clustering within the first 7 years, reflecting the shorter latency of chemotherapy‐related hematologic malignancies relative to radiation‐induced solid tumors [21]. These findings underscore the importance of distinguishing SMN type when interpreting field‐location analyses, as the apparent predominance of out‐of‐field SMNs in this cohort is largely driven by chemotherapy‐related hematologic malignancies rather than reflecting a lower RT‐attributable solid tumor risk.

SMN risk depends on various factors, including patient age, the individual's biological and genetic predisposition, the volume and location of irradiated tissue, the radiation dose, and the combination with CT. RT can induce various forms of DNA damage, including base damage, cross‐links, single‐strand breaks, and double‐strand breaks. DNA repair is crucial for cellular survival, and its efficacy determines the fate of surviving cells and their late sequelae, including genetic and epigenetic mutations or translocations that may lead to radiation carcinogenesis [22]. Many factors may influence radiation‐induced SMNs, including the specific characteristics of the radiation, radiation type (photon versus proton), dose, dose‐rate, dose‐fractionation, dose distribution, scatter dose, and threshold for mutagenicity. Furthermore, CT‐related SMNs can be a sequel of alkylating agents and the epipodophyllotoxin etoposide, most frequently in leukemias. Solid tumors can also be a sequel of alkylating agents, especially when combined with RT [23].

Our findings are consistent with the existing literature, indicating an association between alkylating CT and SMN development. In our cohort, all individuals who developed SMNs had received CT in addition to RT, with the majority receiving alkylating agents, aligning with the Childhood Cancer Survivor Study's demonstration of a dose–response between alkylating agents and SMN rate [13]. We found no significant difference in mean time to SMN between patients who received alkylating versus non‐alkylating CT, though this should be interpreted cautiously given the small number of events. Our results also demonstrate a significantly longer latency for SMNs arising within versus outside the RT field (7.5 vs. 4.0 years), consistent with the known biology of RT‐induced solid tumors and with data from a Canadian multi‐center study reporting a median latency of 24.5 years from RT to sarcoma diagnosis [24]. This latency differential further supports the interpretation that out‐of‐field hematologic malignancies—driven primarily by CT—manifest earlier than RT‐induced solid tumors, and underscores the importance of sustained surveillance across both treatment exposures.

Strengths of this study include its large sample size, especially compared with other cancer studies in LMICs, and its inclusion of outcomes through age 23 years. Additionally, the data come from a single hospital with standard treatment and diagnostic protocols, which may reduce the likelihood that cancer recurrences are misclassified as SMN. While the study was conducted from only one hospital, in 2019, CCHE served nearly half of Egypt's pediatric cancer cases [14].

Limitations of our study include that it includes only CCS who received RT to treat an initial cancer, rather than all CCS, and that the small sample size of CCS who received alkylating CT may lead to insufficient statistical power. While it includes up to 11 years of follow‐up, evidence suggests that SMNs are more likely to occur during or after the second decade following an initial cancer diagnosis, and most established long‐term follow‐up studies in CCS extend up to and beyond 30 years [7, 13]. This relatively short follow‐up duration likely underestimates the true cumulative incidence of SMNs, particularly late‐onset solid tumors, which may not manifest until well into adulthood. Further studies are needed to measure the later occurrence of SMN in CCS cohorts. Additionally, detailed RT parameters (field size and treatment modality) were not captured or analyzed in this study. The absence of this data limits our ability to infer causality between specific RT exposures and SMN development. Our analysis also did not account for known hereditary cancer predisposition syndromes, such as Li‐Fraumeni or RB1‐associated retinoblastoma [25, 26]. Patients with these conditions carry germline mutations that independently confer elevated SMN risk, and their inclusion without stratification may confound estimates of treatment‐related malignancy. Mortality ascertainment relied solely on institutional hospital records, which may introduce underreporting of deaths occurring outside the hospital system. This could result in overestimation of overall survival and underestimation of late mortality in this cohort. Finally, assessment of the proportional hazards assumption revealed violations for most covariates, likely reflecting the limited statistical power associated with the small number of SMN events (*n* = 23) relative to the number of model parameters. Given the small number of observed SMNs, all subgroup analyses should be interpreted with caution as the study was likely underpowered to detect statistically significant differences within these strata.

To our knowledge, this study is one of the first to describe SMN occurrence among CCS. In the MENA region and fills a significant gap in providing recent data from a large LMIC cohort where cancer outcomes, particularly SMN, are rarely reported. Pediatric SMN has been infrequently reported in LMICs, likely due to the absence of effective population‐based registries. One study from Turkey reported only 11 SMNs among 2100 pediatric patients followed for more than 24 years [27]. With CCHE serving nearly half of Egypt's pediatric oncology cases, the patterns observed here are likely representative of a substantial proportion of the national CCS population, yet structured long‐term survivorship care remains limited. The absence of such a registry means that late effects, including SMNs, are likely systematically undercounted. Establishing national registry infrastructure with longitudinal follow‐up capacity is necessary; as pediatric cancer survival rates improve across LMICs, the systems to support those survivors must be built in parallel.

## Author Contributions

**Charlotte L. Sackett:** conceptualization, methodology, writing – review and editing, investigation, writing – original draft, formal analysis. **Mohamed S. Zaghloul:** conceptualization, methodology, investigation, supervision, writing – original draft, writing – review and editing. **Ahmed Aldesouky:** data curation. **Alaa Elhaddad:** data curation. **Li Zhang:** methodology, data curation, writing – review and editing. **Chloe A. Teasdale:** conceptualization, writing – review and editing. **Amr S. Soliman:** conceptualization, methodology, writing – original draft, writing – review and editing.

## Funding

This work was supported by Grant # R25 CA112383 from the National Cancer Institute (PI, Amr Soliman).

## Disclosure

Artificial Intelligence (AI) or other online tools were not used to conduct analyses or write this paper.

## Ethics Statement

This study was approved by the Institutional Review Board (IRB) of the University of Southern California (UP‐21‐00295) and the Scientific Medical Advisory Committee (SMAC) of Children's Cancer Hospital Egypt (CCHE). Dual institutional approval was required because the study was conducted as part of the Cancer Epidemiology Education in Special Populations (CEESP) fellowship program, a collaborative research training initiative funded by the National Cancer Institute. As a CEESP fellow, the lead author, Charlotte Sackett, was enrolled at the University of Southern California, necessitating USC IRB oversight of her research activities. Approval from CCHE's SMAC was additionally required as the institution responsible for patient care and the custodian of the medical records from which study data were abstracted. Patient data were de‐identified prior to analysis, and informed consent was waived in accordance with the policies of both institutions, given the retrospective nature of the study.

## Conflicts of Interest

The authors declare no conflicts of interest.

## Data Availability

The data that support the findings of this study are available on request from the corresponding author. The data are not publicly available due to privacy or ethical restrictions.
